# Expert pianists make specific exaggerations for teaching

**DOI:** 10.1038/s41598-022-25711-3

**Published:** 2022-12-09

**Authors:** Atsuko Tominaga, Günther Knoblich, Natalie Sebanz

**Affiliations:** Department of Cognitive Science, Central European University, Quellenstraße 51, 1100 Vienna, Austria

**Keywords:** Psychology, Human behaviour

## Abstract

Experts modulate their performance of actions for teaching purposes, performing slower and exaggerated movements when demonstrating novel actions to novices. The present study asked whether such modulations also occur during teaching performance of a music instrument, where subtle movement modulations are crucial for achieving artistic expression. While exaggerating performances of goal-directed actions outside of artistic contexts may be straightforward, it is an open question whether and how exaggeration for the purpose of teaching operates for actions that are expressive even when performed outside of a teaching context. Pianists were asked to demonstrate to students the techniques required for implementing notated expressions, compared to performing the piece without didactic intentions. Expressions in the piece concerned either articulation (i.e., legato and staccato) or dynamics (i.e., forte and piano). The pianists played either with the goal to perform the piece to an audience or with the goal to teach the respective techniques to novices. When intending to teach articulation, skilled pianists produced more exaggerated staccato. When intending to teach dynamics, they created a larger contrast between forte and piano. We found consistent results across a simple musical scale (Experiment 1) and a more naturalistic piece of music (Experiment 2). These findings show that teaching-specific action modulations generalise to expressive actions and suggest that action modulations serve to highlight the most relevant aspects of the actions to be learnt.

## Introduction

Deliberate teaching has supported human skill transmission over generations and provides a key route for learning^1,2^. Experts modulate their behaviour so that novices can extract relevant information to learn a novel skill. For instance, adults modulate their speech (motherese) and actions (motionese) when demonstrating a skill to infant learners^3,4^. These modulations include slowing down and exaggerating sounds and actions. Similar findings were obtained in studies with adult learners, where exaggerations were observed when native British English speakers were talking to second language English learners^5^ and when skilled adults were teaching xylophone melodies to novices^6^. The observed modulations are thought to provide communicative signals that can facilitate learning by affecting attention and memory^7^.

Previous research has shown that demonstrators not only adjust their gestures^8^ and actions^9^ to learners’ skills but engage in specific action modulations to highlight certain aspects of demonstrated actions. For example, Schaik and colleagues^10^ showed that adults used specific action modulations for demonstrating different action effects of objects to infants. Ho and colleagues^11^ found that demonstrators chose costly movement paths at structurally important points to disambiguate one goal over other possibilities.

Performing actions with exaggeration is straightforward for actions that are normally performed in the most efficient way possible^12^. However, how can particular aspects of actions be highlighted when the actions themselves are expressive even outside of a teaching context? This is the case in music performance, where pieces are played with expression. Expressivity is a vital component of performance and typically the main focus of music teaching^13^. Expressive skills are generally considered to be separate from technical skills, however, fine motor control is required to implement subtle sound modulations in expressive performance^14^. In some music genres such as Western classical music, it is crucial to acquire the motor skills needed to perform a piece expressively. For example, pianists translate their own interpretations of music and convey their emotions by modulating specific parameters such as timing, smoothness and loudness of sound. This raises the question of whether and how musicians modulate their actions during didactic demonstration of expressive techniques.

In naturalistic teaching settings, teachers have many possibilities for how to convey to a learner how to play a piece expressively. They may use verbal communication^15,16^ and nonverbal bodily cues^17^ as well as modulating the sounds that they are producing. In the present study, we focused entirely on the teaching of expressive techniques through sound in order to determine whether expert musicians would systematically exaggerate the actions necessary to implement a particular technique for musical expression. Pianists were asked to perform a piece to demonstrate to a learner how to implement the notated expressions (teaching condition) and to play the same piece for an audience (performing condition). Two basic expressive techniques in piano performance were used: articulation (legato and staccato) and dynamics (forte and piano). Articulation was assessed by key-overlap time between two consecutive notes. Positive key-overlap time indicates legato styles whereas negative key-overlap time indicates staccato styles. Dynamics was evaluated by key velocity of each key press. Higher key velocity means forte styles while lower key velocity means piano styles.

If experts rely on generic action modulations, it can be expected that they will play more slowly during didactic demonstration, regardless of the kind of techniques to be taught. To the extent that experts use action modulations to support their teaching of specific techniques, they should exaggerate legato (i.e., key-overlap time should be more positive) and staccato (i.e., key-overlap time should be more negative) when teaching articulation whereas they should exaggerate forte (i.e., key velocity should be higher) and piano (i.e., key velocity should be lower) when teaching dynamics. Furthermore, one could speculate that they might produce modulations specifically at structurally important points that best highlight the technique to be taught. Importantly, they should avoid modulating irrelevant properties of expression (e.g., the smoothness of sound while teaching dynamics).

In Experiment 1, we employed a simple musical scale to examine whether and how skilled pianists vary their performance depending on which expressive techniques (i.e., either articulation or dynamics) they are teaching. Experiment 2 was conducted to replicate our findings from Experiment 1 with a more naturalistic piece of music.

## Experiment 1

### Materials and methods

#### Participants

We recruited 36 piano experts who played the piano for at least the past 10 years or were studying advanced piano performance at a music school at the time of recruitment. For data analysis, we excluded three participants due to experimental errors, and two participants because they deviated substantially from the prescribed tempo (outside 2 standard deviations from the average tempo across participants). Thirty-one participants (15 female) were included in data analysis. Most participants were right-handed (left: 2, ambidextrous: 2) with a mean age of 24.16 (*SD* = 4.26). They had 12.45 years of practice on average (*SD* = 5.66). 10 participants had experience in teaching the piano (*M* = 3.48 years, *SD* = 3.51). All participants gave their informed consent before the experiment started and received vouchers for their participation. The study (No. 2018-124) was approved by the United Ethical Review Committee for Research in Psychology (EPKEB) in Hungary. The experiment was carried out in accordance with relevant guidelines and regulations.

#### Apparatus and stimuli

A weighted Yamaha MIDI digital piano was used to record participants’ performance via Max/MSP (https://cycling74.com/products/max) on a MacBook Pro with Mac OS X Mojave 10.14.3. The laptop and piano were connected to a high-fidelity soundcard (Focusrite Scarlett 6i6) to deliver a metronome and piano sound. All auditory feedback was given to participants through headphones (Audio-Technica ATH-M50X). Sheet music was displayed on a computer monitor in front of the participants. The pitch, onset and offset time of each note, and key velocity profiles were obtained from MIDI data using Max/MSP patchers.

**Figure 1:**
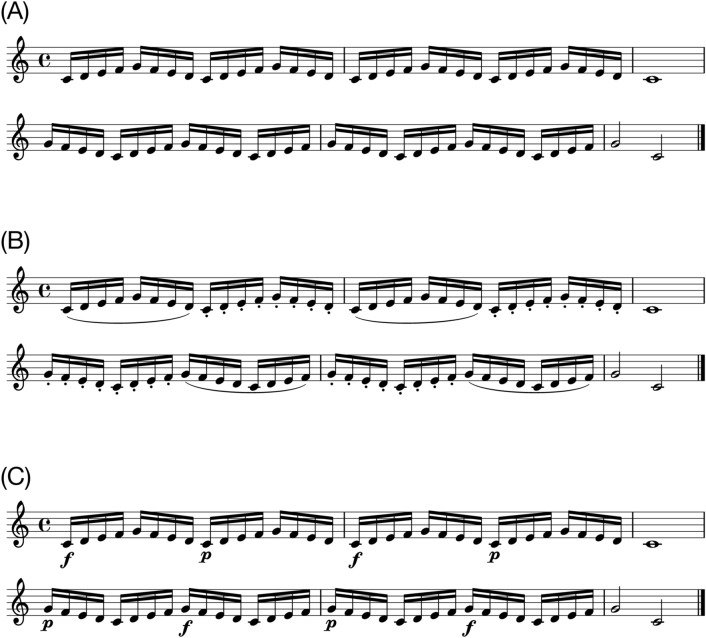
(**A**) Original sheet music. (**B**) Articulation. The curved line (slur) indicates legato and the dots indicate staccato. (**C**) Dynamics. The symbol ‘f’ denotes forte and the symbol ‘p’ denotes piano. For data analysis, only the 16th notes were included.

One musical excerpt was used as a stimulus. The excerpt was taken from “A Dozen a Day—Play with Ease in Many Keys” by Edna-Mae Burnam and modified for the experiment. It consisted of a 6-measure isochronous melody noted in a 4/4 metre. The stimulus was composed in C major to be played with the right hand only. Original sheet music (i.e., sheet music without expressive notations, Fig. 1A) was used for the purpose of practice. Expressive notations were added to the original sheet music for the experiment. They referred to either articulation or dynamics (Fig. 1B,C). Articulation was notated as either legato or staccato. Legato indicates that musical notes are to be connected and should sound smooth. Staccato requires producing musical notes with shortened duration, keeping them separate from each other. Dynamics was notated as either forte or piano. Forte indicates that musical notes should be played loudly whereas piano indicates that musical notes should be played softly. The notation did not include any indication of fingering (i.e., the positioning of the fingers when playing the piano) because the piece was simple and pilot testing had shown that specifying fingering was not necessary.

#### Procedure

First, participants were allocated to either the teaching or performing condition and asked to practise the excerpt with the notated expression of either articulation or dynamics (Fig. 1B,C). As soon as they had produced the excerpt with the notated expression without pitch errors twice consecutively, the test trials began.

In the teaching condition, participants were instructed to play the excerpt of music as if they were teaching it to students. It was mentioned that the students already knew the sequence of the tones and that they were trying to learn how to perform the piece with the notated expression by listening to the participant’s performance. In the performing condition, participants were asked to play the excerpt of music as if they were performing it to an audience (see details in Supplementary Material 1). Participants played the piece 8 times per technique per condition, so there were 32 trials in total (2 conditions × 2 techniques × 8 trials). The order of the conditions was blocked and counterbalanced across participants. The order of the techniques within each condition was also blocked and counterbalanced across participants. A leading metronome (80 quarter beats per minute, 8 beats) indicated the target tempo before each trial.

At the end of the experiment, participants filled in a questionnaire asking about their demographic information and experience in piano performance/teaching.

#### Data analysis

Three dependent variables were computed for data analysis. Inter-onset intervals (IOIs) are the intervals between onsets of adjacent notes and provide a measure of tempo. Key-overlap time (KOT) is the difference between the offset time of the current tone (i.e., key release time) and the onset time of the ensuing tone and is a measure for the smoothness of musical sequences^18^. A positive value indicates smooth legato styles due to overlap between the current and ensuing tone whereas a negative value indicates sharp staccato styles due to separation between the current and ensuing tone. Tone intensity is assessed by key velocity (KV) and measures the loudness of a musical note. A higher value indicates forte styles whereas a lower value indicates piano styles. The value of KV in MIDI varies between 0 (minimum) and 127 (maximum).

Data cleaning, preprocessing and statistical analysis were performed in R version 4.0.5. For statistical analysis, only 16th notes with expressive notations were included. Overall, five trials were excluded from data analysis because participants did not follow the sheet music or stopped performing before the end. Pitch errors were identified by comparing the sequence of musical notes produced by a participant with the sequence of musical notes according to the sheet music. Pitch errors included either extra, missing or substituted tones and were manually removed by using the *editData* R package. For onsets, 11.65% of the trials contained at least one pitch error (extra notes: 6.28%, missing notes: 5.07%, substituted notes: 0.30%). For offsets, 14.08% of the trials contained at least one pitch error (extra notes: 6.28%, missing notes: 5.17%, substituted notes: 2.63%). We found that some participants did not precisely follow the sheet music (e.g., they held some notes longer than notated), therefore the order of offsets did not correspond to that of onsets. We counted these as errors and removed the erroneous notes even if the order of onsets was correct. As a result, less than 1% of total responses were corrected. In addition to pitch errors, we removed outliers for IOIs, KOT and KV, defined as values more than 3 standard deviations from the mean of each dependent variable. For each dependent variable, this resulted in less than 5% of overall responses being removed as outliers.

We performed separate analyses for the two techniques (i.e., articulation and dynamics). A paired-sample *t*-test or a Wilcoxon Signed-rank test (a non-parametric alternative to a paired *t*-test) was performed to compare the mean IOIs in the teaching and performing condition. For KOT and KV, we performed a 2 × 2 repeated-measures analysis of variance (ANOVA) with the factors Condition (teaching vs. performing) and Subcomponent (Articulation: legato vs. staccato or Dynamics: forte vs. piano, respectively). The *t.test* or *wilcox.test* function in the *stats* R package was used for a *t*-test or a Wilcoxon Signed-rank test. For calculating an effect size, we used the *rstatix* R package. The *aov_car* function in the *afex* R package was used for a repeated-measures ANOVA. For post-hoc comparisons on the estimated marginal means, we used the *emmeans* R package.

### Results

All effects are reported as significant at *p* < 0.05. We first report results for performance of the piece with the notated expression of articulation (Fig. 1B), followed by performance of the piece with the notated expression of dynamics (Fig. 1C). To recall our predictions, if participants play more slowly when they are trying to teach, inter-onset intervals (IOIs) should be larger when teaching. If participants specifically modulate relevant aspects of the techniques they are trying to teach, key-overlap time (KOT) should be more positive for legato and more negative for staccato when teaching articulation, and key velocity (KV) should be higher for forte and lower for piano when teaching dynamics.

### Articulation

#### Inter-onset intervals (IOIs)

**Figure 2:**
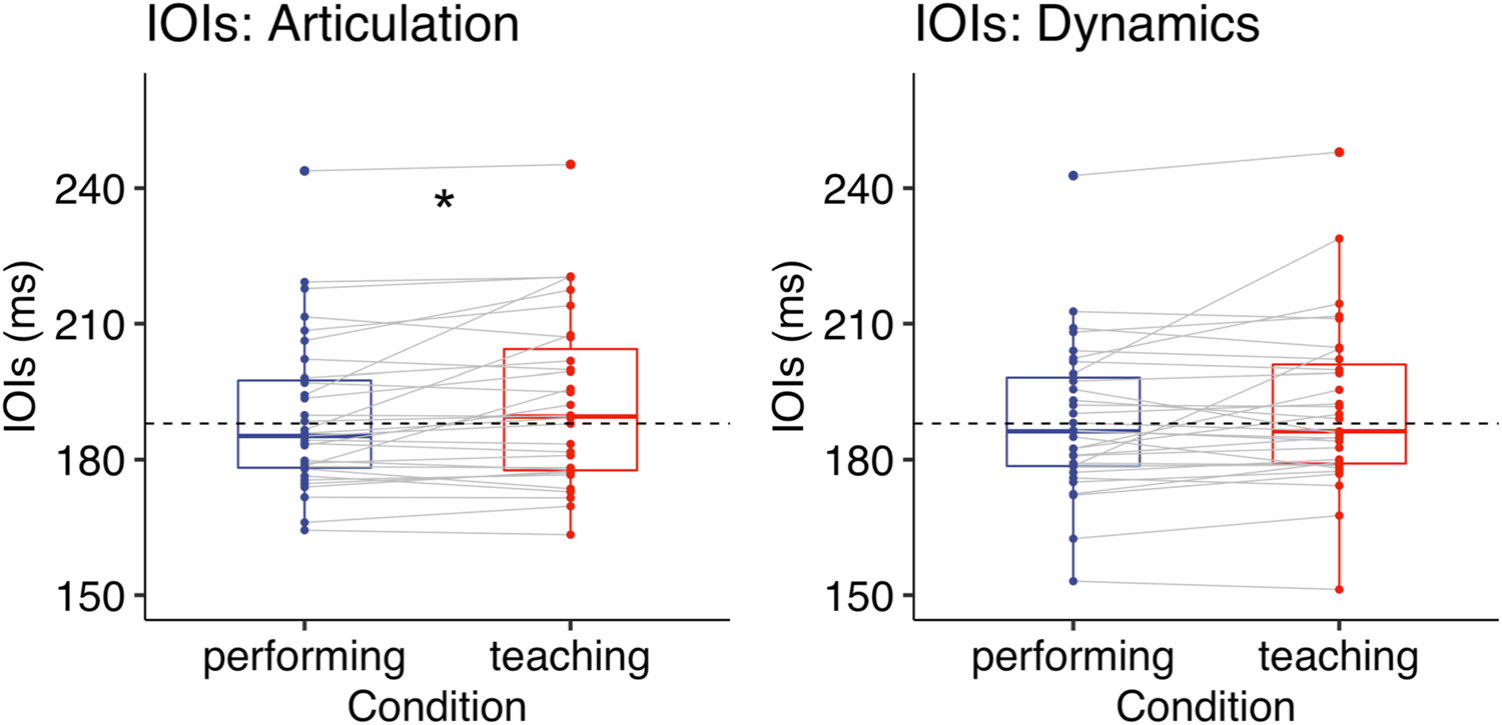
Experiment 1: IOIs (ms) when playing the piece with either articulation (left) or dynamics (right). A dashed line represents the tempo given by a metronome. Each box indicates the IQR with the median, and whiskers extend to a maximum of 1.5 × IQR beyond the box. Significance levels: * < 0.05, ** < 0.01, *** < 0.001.

To compare the mean IOIs between the teaching and performing condition, we conducted a Wilcoxon Signed-rank test, instead of a paired *t*-test, because a Shapiro–Wilk test showed that the distribution of the mean difference was significantly different from the normal distribution (*p* < 0.001). The Wilcoxon Signed-rank test revealed that participants played more slowly in the teaching condition [*Mdn* = 189.55 (ms), *IQR* = 26.74] than in the performing condition [*Mdn* = 185.23 (ms), *IQR* = 19.30] while playing the piece with the notated articulation (*p* = 0.021, *r* = 0.41, two-tailed, Fig. 2).

#### Key-overlap time (KOT)

**Figure 3:**
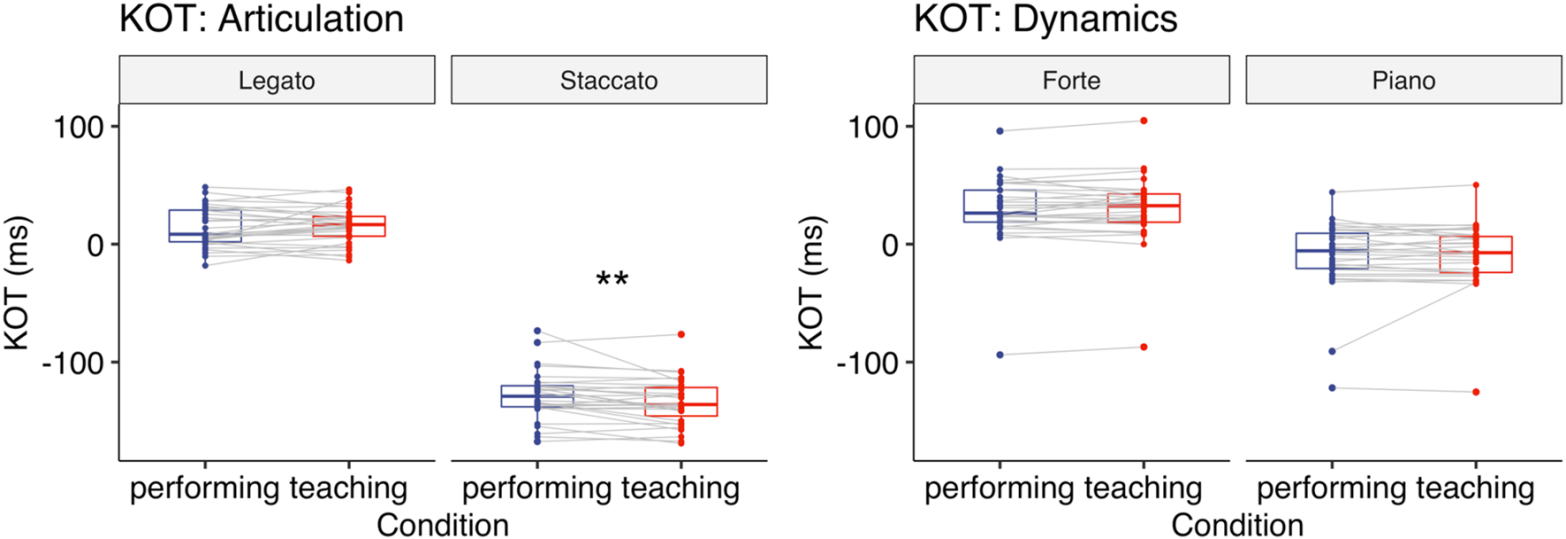
Experiment 1: KOT (ms) when playing the piece with either articulation (left) or dynamics (right). Each box indicates the IQR with the median, and whiskers extend to a maximum of 1.5 × IQR beyond the box. Significance levels: * < 0.05, ** < 0.01, *** < 0.001.

A two-way repeated-measures ANOVA with the factors Condition (teaching vs. performing) and Articulation (legato vs. staccato) revealed that there was a significant main effect of Articulation (*F*(1,30) = 1149, *p* < 0.001, ${\eta }_{G}^{2}$ = 0.94) and a significant interaction between Condition and Articulation (*F*(1,30) = 8.52, *p* = 0.007, ${\eta }_{G}^{2}$ = 0.010, Fig. 3). Post-hoc comparisons based on the estimated marginal means with Tukey adjustment showed that for staccato KOT was more negative in the teaching condition [*M* = − 133.79 (ms), *SD* = 20.13] than in the performing condition [*M* = − 128.34 (ms), *SD* = 20.86] (*p* = 0.005). For legato there was no significant difference in KOT between the teaching [*M* = 15.90 (ms), *SD* = 15.51] and performing condition [*M* = 14.01 (ms), *SD* = 17.02] (*p* = 0.32).

#### Key velocity (KV)

**Figure 4:**
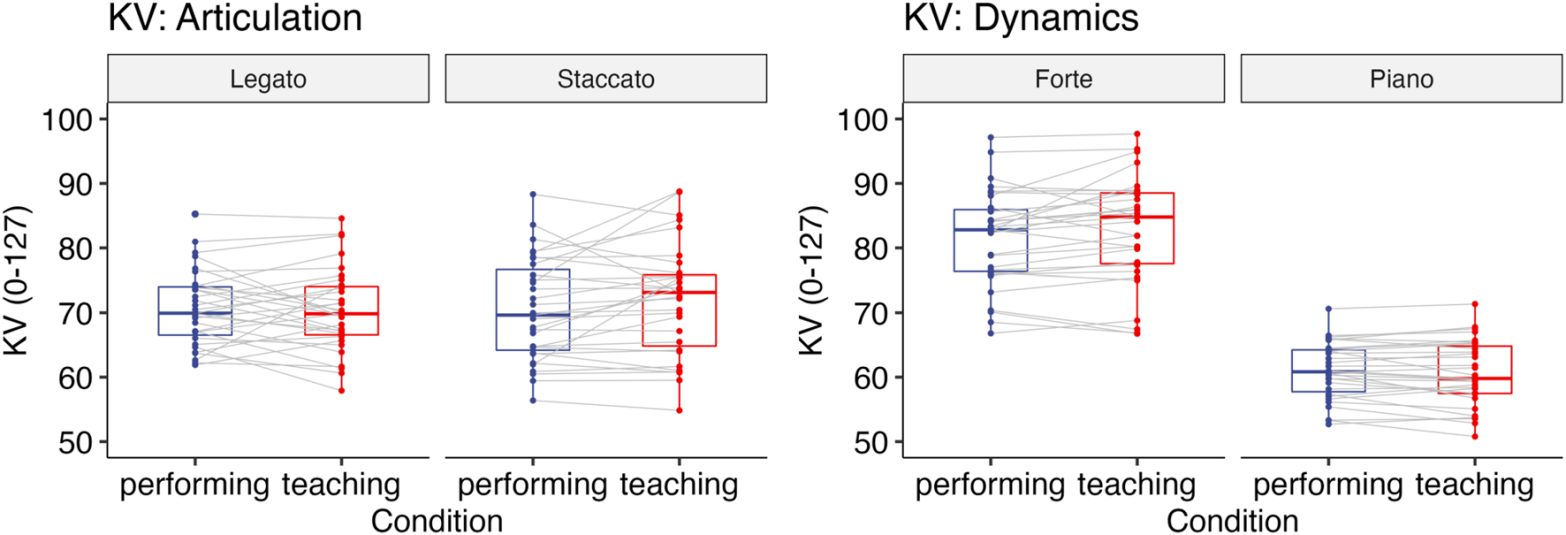
Experiment 1: KV (0–127) when playing the piece with either articulation (left) or dynamics (right). Each box indicates the IQR with the median, and whiskers extend to a maximum of 1.5 × IQR beyond the box. Significance levels: * < 0.05, ** < 0.01, *** < 0.001.

A two-way repeated-measures ANOVA with the factors Condition (teaching vs. performing) and Articulation (legato vs. staccato) showed that there was a significant interaction between Condition and Articulation (*F*(1,30) = 7.45, *p* = 0.011, ${\eta }_{G}^{2}$ = 0.004, Fig. 4). However, post-hoc comparisons based on the estimated marginal means with Tukey adjustment did not find a significant difference between the teaching [Legato: *M* = 70.34, *SD* = 6.46; Staccato: *M* = 72.00, *SD* = 8.74] and performing condition [Legato: *M* = 70.68, *SD* = 5.86, Staccato: *M* = 70.41, *SD* = 7.97] for each subcomponent (Legato: *p* = 0.68, Staccato: *p* = 0.09).

### Dynamics

#### Inter-onset intervals (IOIs)

To compare the mean IOIs between the teaching and performing condition, we conducted a Wilcoxon Signed-rank test, instead of a paired *t*-test, because a Shapiro–Wilk test showed that the distribution of the mean difference was significantly different from the normal distribution (*p* < 0.001). The Wilcoxon Signed-rank test revealed no significant difference between the teaching condition [*Mdn* = 186.28 (ms), *IQR* = 21.83] and the performing condition [*Mdn* = 186.27 (ms), *IQR* = 19.59] while playing the piece with the notated dynamics (*p* = 0.11, *r* = 0.29, two-tailed, Fig. 2).

#### Key velocity (KV)

A two-way repeated-measures ANOVA with the factors Condition (teaching vs. performing) and Dynamics (forte vs. piano) showed that there was a significant main effect of Dynamics (*F*(1,30) = 369, *p* < 0.001, ${\eta }_{G}^{2}$ = 0.74) and a significant interaction between Condition and Dynamics (*F*(1,30) = 6.83, *p* = 0.014, ${\eta }_{G}^{2}$ = 0.003, Fig. 4). However, post-hoc comparisons based on the estimated marginal means with Tukey adjustment did not find a significant difference between the teaching [Forte: *M* = 82.78, *SD* = 8.31, Piano: *M* = 60.63, *SD* = 5.08] and performing condition [Forte: *M* = 81.65, *SD* = 7.43, Piano: *M* = 60.99, *SD* = 4.41] for each subcomponent (Forte: *p* = 0.068, Piano: *p* = 0.24).

#### KV transition points

Additionally, we conducted an exploratory analysis by focusing on specific points at which each subcomponent changed from one to the other (i.e., forte to piano: FtoP, piano to forte: PtoF). These points could be structurally important to make a contrast between forte and piano. We calculated the KV difference for each interval by subtracting the KV value of the current note from that of the following note. Outliers were removed using the same criteria as for the other dependent variables and less than 5% of the data were excluded from analysis. A two-way repeated-measures ANOVA with the factors Condition (teaching vs. performing) and Transition Type (FtoP vs. PtoF) showed that there was a significant main effect of Transition Type (*F*(1,30) = 224, *p* < 0.001, ${\eta }_{G}^{2}$ = 0.83) and a significant interaction between Condition and Transition Type (*F*(1,30) = 25.99, *p* < 0.001, ${\eta }_{G}^{2}$ = 0.033, Fig. 9). Post-hoc comparisons based on the estimated marginal means with Tukey adjustment showed that there was a larger KV difference when changing from forte to piano (*p* = 0.002) and from piano to forte (*p* < 0.001) in the teaching condition [FtoP: *M* = − 14.11, *SD* = 7.17; PtoF: *M* = 24.27, *SD* = 9.77] than in the performing condition [FtoP: *M* = − 11.70, *SD* = 5.99, PtoF: *M* = 20.87, *SD* = 8.67].

#### Key-overlap time (KOT)

A two-way repeated-measures ANOVA with the factors Condition (teaching vs. performing) and Dynamics (forte vs. piano) revealed that there was a significant main effect of Dynamics (*F*(1,30) = 308, *p* < 0.001, ${\eta }_{G}^{2}$ = 0.30, Fig. 3), reflecting more key-overlap for forte notes compared to piano notes. Neither the main effect of Condition (*F*(1,30) = 0.60, *p* = 0.44, ${\eta }_{G}^{2}$ = 0.000) nor the interaction between Condition and Dynamics (*F*(1,30) = 0.053, *p* = 0.82, ${\eta }_{G}^{2}$ = 0.000) was significant.

### Discussion

The findings from Experiment 1 indicated that skilled pianists modified their performance for teaching purposes. For IOIs, we found a small but significant slowing down during teaching specifically when playing the piece with the notated articulation. This finding is in line with earlier studies that found slower performance of actions in a teaching context^e.g.,^^3,6^. However, we did not find a significant difference in tempo when participants were teaching dynamics. It could be that trying to keep the prescribed tempo (conveyed through the leading metronome) limited the extent to which participants slowed down their performance during teaching. Another possibility is that slower performance may be beneficial to highlight the relation between two notes (i.e., to what extent two notes overlap) and was therefore employed when teaching articulation, whereas slower performance might not help when teaching dynamics.

The results for KOT and KV showed that participants successfully highlighted relevant aspects of articulation and dynamics. Specifically, participants exaggerated staccato when teaching articulation. Moreover, our exploratory analysis demonstrated that participants made a larger contrast in dynamics at transition points (i.e., forte to piano or piano to forte). Importantly, participants did not modulate their performance in terms of irrelevant aspects of the techniques for teaching purposes (e.g., modulating the smoothness of sound while teaching dynamics). These findings confirmed that participants modulated their performance in systematic and fine-grained ways to teach particular techniques.

## Experiment 2

In Experiment 1, we employed a simple musical scale to maximise experimental control. To test whether our results generalise to a more naturalistic piece, in Experiment 2, we chose an actual piano piece and modified it for the purpose of the experiment. If pianists selectively highlight the relevant aspects of the techniques to be taught also in a more naturalistic piece containing more opportunities for expression, key-overlap time should be again more positive for legato and more negative for staccato while teaching articulation. When teaching dynamics, key velocity should be higher for forte and lower for piano. Given the findings we observed in Experiment 1, we also predicted that participants would make a larger key velocity contrast between forte and piano at transition points, and that they might play more slowly when teaching, especially when teaching articulation.

### Materials and methods

#### Participants

We recruited 21 piano experts who already had a degree (above bachelor or equivalent) in piano performance/teaching or were studying advanced piano performance at a music school at the time of recruitment. For data analysis, we excluded one participant due to insufficient motor skills. Twenty participants (9 female) were included in the data analysis. Most participants were right-handed (left: 2) with a mean age of 25.90 (*SD* = 4.68). They had 15.65 years of practice on average (*SD* = 5.67). 11 participants had experience in teaching the piano (*M* = 4.14 years, *SD* = 3.65). All participants gave their informed consent before the experiment started and received vouchers for their participation. The study (No. 2018-124) was approved by the United Ethical Review Committee for Research in Psychology (EPKEB) in Hungary. The experiment was carried out in accordance with relevant guidelines and regulations.

#### Apparatus and stimuli

**Figure 5:**
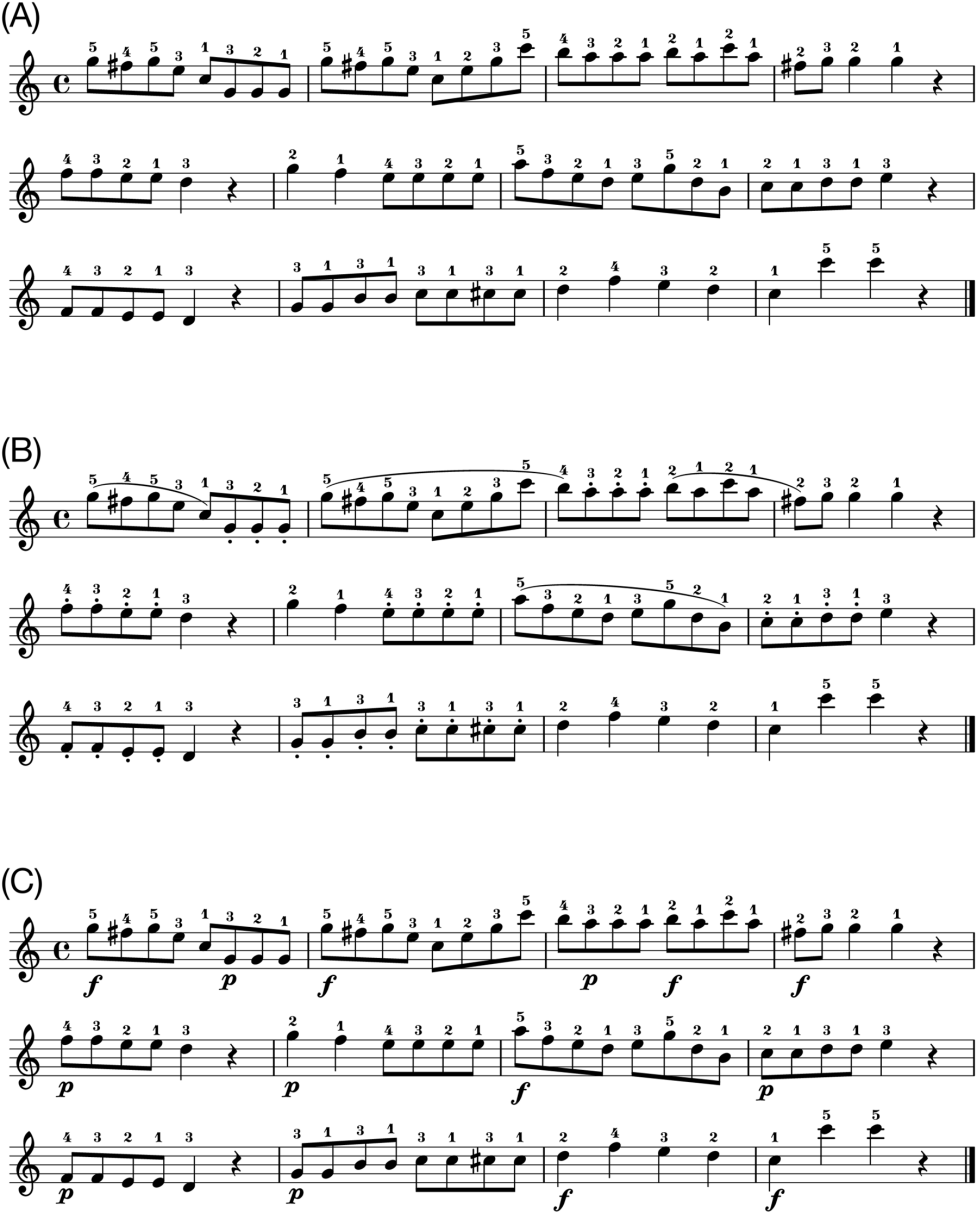
(**A**) Original sheet music. (**B**) Articulation. The curved line (slur) indicates legato and the dots indicate staccato. (**C**) Dynamics. The symbol ‘f’ denotes forte and the symbol ‘p’ denotes piano. For data analysis, only the 8th notes were included.

The same apparatus as in Experiment 1 was used. We selected Clementi’s Sonatina Op.36 (No. 3) in C major as a stimulus because it contains our targeted expressions (i.e., articulation and dynamics) and is relatively simple in terms of motor skills. The first 12 measures of the original piece were used and modified so that the piece had an almost equal number of data points for each dependent variable. The modified piece consisted of a 12-measure isochronous melody notated in a 4/4 metre to be played with the right hand only. Original sheet music was used for the purpose of practice (Fig. 5A). Expressive notations were added to the original sheet music for the experiment (Fig. 5B,C). These excerpts were confirmed to be musically natural by a doctoral student in piano performance at Liszt Ferenc Academy of Music in Hungary. The fingering was also assigned and confirmed by the same doctoral student.

#### Procedure

We employed the same procedure as in Experiment 1 with several modifications. First, all participants were required to memorise the piece without expressive notations (i.e., Fig. 5A) prior to the experiment so that they had enough time to familiarise themselves with the piece and perform it without pitch errors while performing it with notated expressions in the lab. Second, we modified the wording of instructions for the performing condition so that both instructions had the same focus on expressive notations because there had been less emphasis on expression in the instruction in the performing condition in Experiment 1 (see details in Supplementary Material 1). Third, participants could choose their preferred tempo from one of three options (100, 110 and 120 quarter beats per minute) because some evidence shows that musicians have their own preferred tempo, which affects their temporal variability^19^. The chosen tempo was again cued by a leading metronome. To make sure that participants memorised the piece and had sufficient motor skills, we asked participants to perform the piece without looking at the original sheet music (Fig. 5A). Those who could not perform the piece without pitch errors twice consecutively within five attempts were excluded from data analysis (as a result, one participant was excluded). The rest of the procedure was identical to Experiment 1.

#### Data analysis

Data cleaning, preprocessing and statistical analysis were almost identical to Experiment 1. For statistical analysis, only 8th notes with expressive notations were included. As a result, only one 8th note in the 4th measure without any expression was not included. IOIs were normalised by their preferred tempo because each participant chose a tempo from the three options. Given the different tempi, key-overlap ratios (KORs) were calculated by dividing KOT by the mean IOI of each performance to normalise KOT. Additionally, we included KV difference (i.e., KV difference for each interval) at transition points (i.e., forte to piano or piano to forte) as a dependent variable based on the findings of Experiment 1. Three trials were entirely excluded from data analysis because participants did not follow the sheet music closely enough. Using the same approach as in Experiment 1, pitch errors were removed manually. For onsets, 11.62% of the trials contained at least one pitch error (extra notes: 5.81%, missing notes: 5.49%, substituted notes: 0.31%). For offsets, 17.90% of the trials contained at least one pitch error (extra notes: 5.81%, missing notes: 5.49%, substituted notes: 6.59%). As a result, less than 1% of total responses were corrected. For each dependent variable, removing outliers (i.e., responses outside 3 standard deviations from the mean) resulted in less than 5% of overall responses being removed.

### Results

As Experiment 1, we first report results for performance of the piece with the notated expression of articulation (Fig. 5B), followed by performance of the piece with the notated expression of dynamics (Fig. 5C).

### Articulation

#### Inter-onset intervals (IOIs)

**Figure 6:**
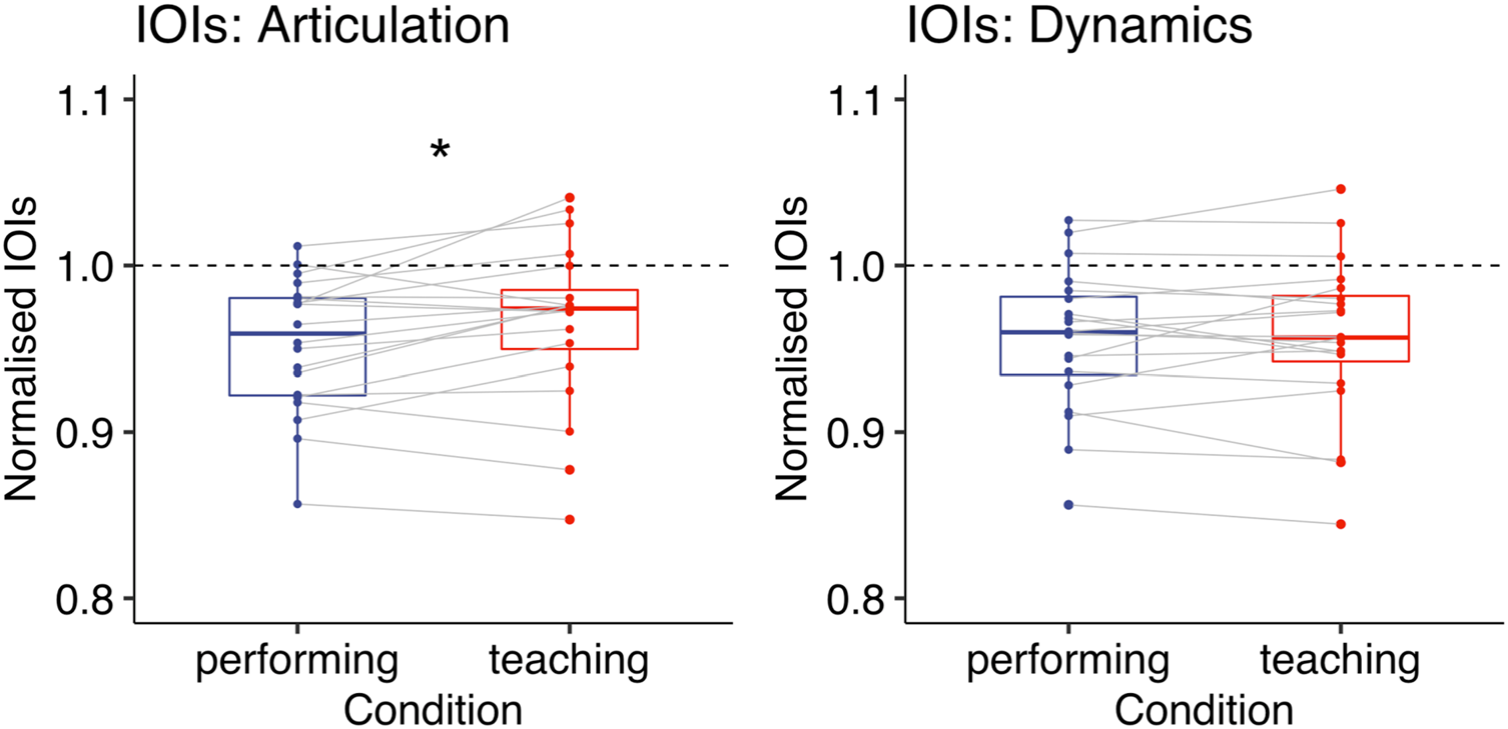
Experiment 2: Normalised IOIs when playing the piece with either articulation (left) or dynamics (right). A dashed line represents the tempo given by a metronome. Each box indicates the IQR with the median, and whiskers extend to a maximum of 1.5 × IQR beyond the box. Significance levels: * < 0.05, ** < 0.01, *** < 0.001.

A paired-sample *t*-test showed that participants played more slowly in the teaching condition [*M* = 0.97, *SD* = 0.049] than in the performing condition [*M* = 0.95, *SD* = 0.040] while playing the piece with the notated articulation (*t*(19) = 2.47, *p* = 0.023, Cohen’s *d* = 0.55, two-tailed, Fig. 6). A Wilcoxon Signed-rank test also confirmed that there was a significant difference between the two conditions (*p* = 0.033, *r* = 0.48, two-tailed).

#### Key-overlap ratios (KORs)

**Figure 7:**
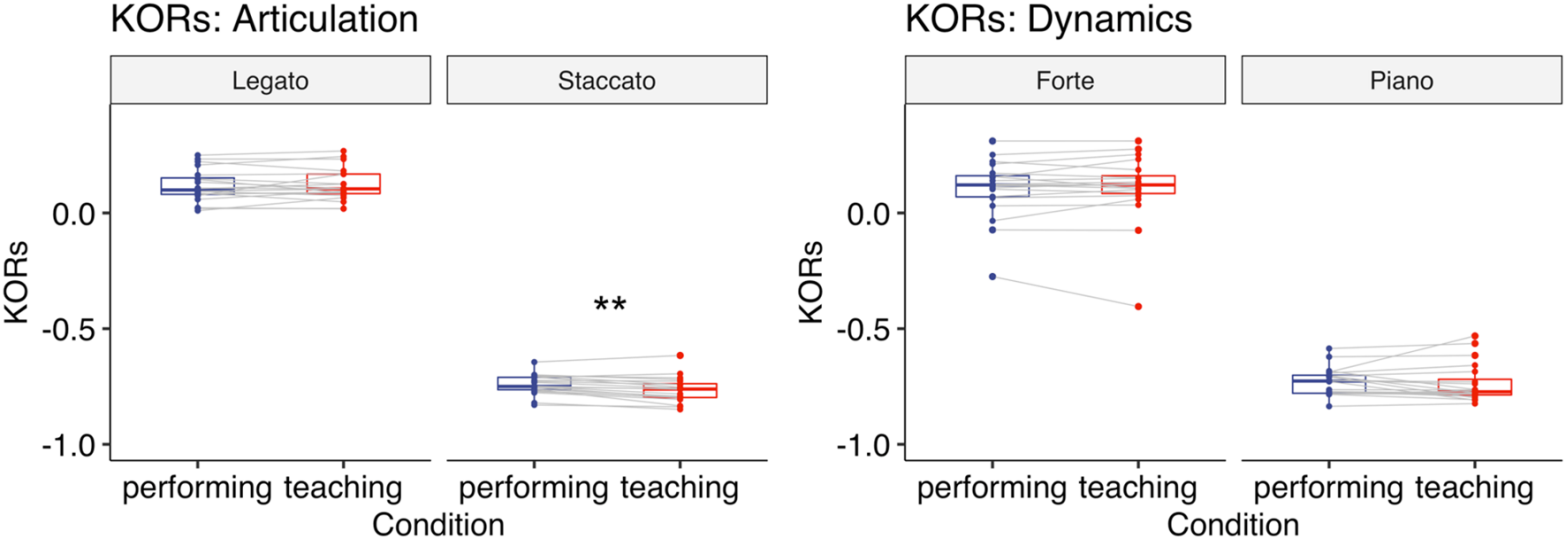
Experiment 2: KORs when playing the piece with either articulation (left) or dynamics (right). Each box indicates the IQR with the median, and whiskers extend to a maximum of 1.5 × IQR beyond the box. Significance levels: * < 0.05, ** < 0.01, *** < 0.001.

A two-way repeated-measures ANOVA with the factors Condition (teaching vs. performing) and Articulation (legato vs. staccato) showed that there was a significant main effect of Articulation (*F*(1,19) = 2566, *p* < 0.001, ${\eta }_{G}^{2}$ = 0.98) and a significant interaction between Condition and Articulation (*F*(1,19) = 8.75, *p* = 0.008, ${\eta }_{G}^{2}$ = 0.01, Fig. 7). Post-hoc comparisons based on the estimated marginal means with Tukey adjustment showed that for staccato KORs were more negative in the teaching condition [*M* = **− **0.76, *SD* = 0.06] than in the performing condition [*M* = − 0.74, *SD* = 0.04] (*p* =  < 0.001). For legato there was no significant difference in KORs between the teaching [*M* = 0.12, *SD* = 0.07] and performing condition [*M* = 0.12, *SD* = 0.07] (*p* = 0.64).

#### Key velocity (KV)

**Figure 8:**
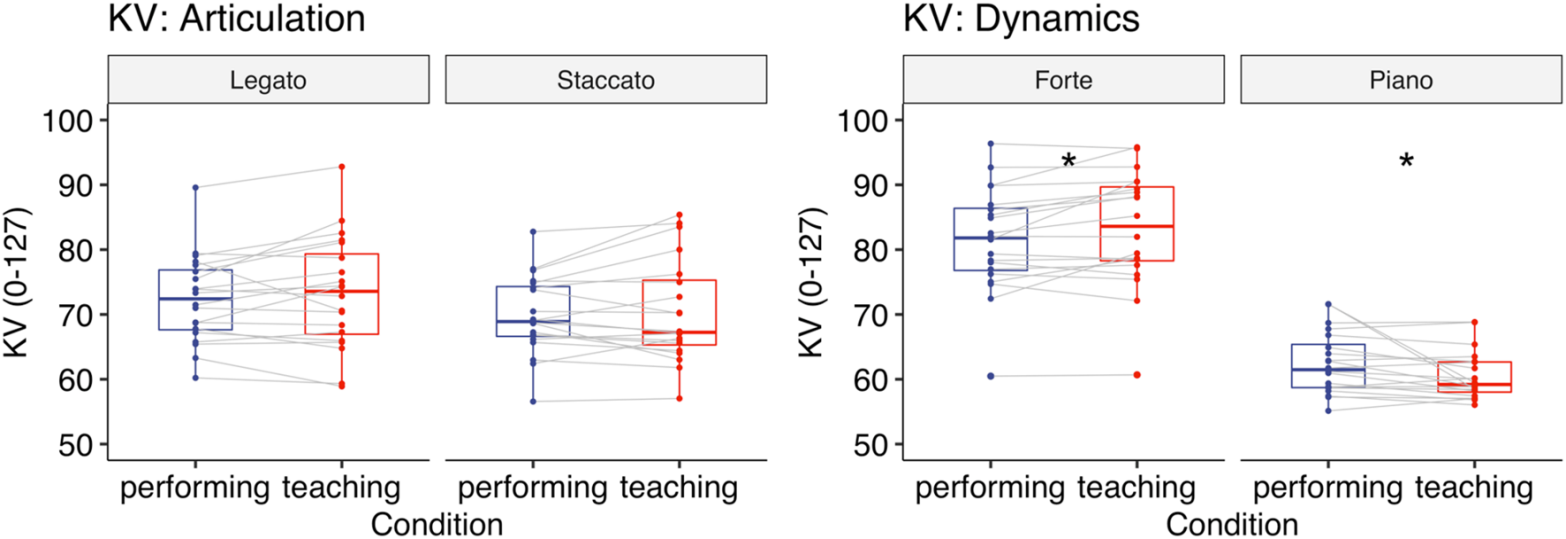
Experiment 2: KV (0–127) when playing the piece with either articulation (left) or dynamics (right). Each box indicates the IQR with the median, and whiskers extend to a maximum of 1.5 × IQR beyond the box. Significance levels: * < 0.05, ** < 0.01, *** < 0.001.

A two-way repeated-measures ANOVA with the factors Condition (teaching vs. performing) and Articulation (legato vs. staccato) showed that there was a significant main effect of Articulation (*F*(1,19) = 7.85, *p* = 0.011, ${\eta }_{G}^{2}$ = 0.03, Fig. 8), reflecting louder sound for legato notes compared to staccato notes. Neither the main effect of Condition (*F*(1,19) = 1.34, *p* = 0.26, ${\eta }_{G}^{2}$ = 0.003) nor the interaction between Condition and Articulation (*F*(1,19) = 0.15, *p* = 0.71, ${\eta }_{G}^{2}$ = 0.000) was significant.

### Dynamics

#### Inter-onset intervals (IOIs)

A paired-sample *t*-test showed no significant difference between the teaching condition [*M* = 0.96, *SD* = 0.048] and the performing condition [*M* = 0.96, *SD* = 0.043] while playing the piece with the notated dynamics (*t*(19) = 0.21, *p* = 0.84, Cohen’s *d* = 0.05, two-tailed, Fig. 6). A Wilcoxon Signed-rank test also confirmed that there was no significant difference between the two conditions (*p* = 0.96, *r* = 0.02, two-tailed).

#### Key velocity (KV)

A two-way repeated-measures ANOVA with the factors Condition (teaching vs. performing) and Dynamics (forte vs. piano) showed that there was a significant main effect of Dynamics (*F*(1,19) = 131, *p* < 0.001, ${\eta }_{G}^{2}$ = 0.72) and a significant interaction between Condition and Dynamics (*F*(1,19) = 6.11, *p* = 0.023, ${\eta }_{G}^{2}$ = 0.018, Fig. 8). Post-hoc comparisons based on the estimated marginal means with Tukey adjustment revealed that KV was higher in the teaching condition [*M* = 83.20, *SD* = 8.84] than in the performing condition [*M* = 81.49, *SD* = 8.12] (*p* = 0.020) when instructed to play forte. Also, KV was lower in the teaching condition [*M* = 60.54, *SD* = 3.76] than in the performing condition [*M* = 62.43, *SD* = 4.82] (*p* = 0.046) when instructed to play piano.

#### KV transition points

**Figure 9:**
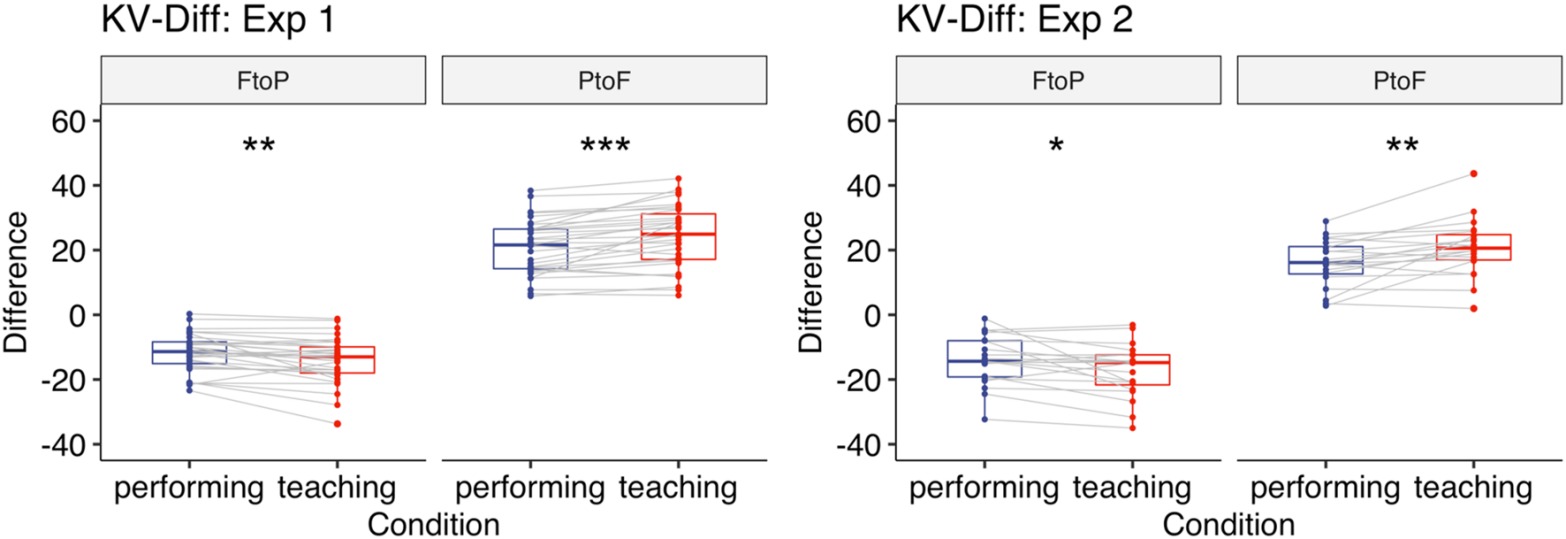
Experiment 1, 2: KV Difference at transition points when playing the piece with dynamics (left: Experiment 1, right: Experiment 2). Each box indicates the IQR with the median, and whiskers extend to a maximum of 1.5 × IQR beyond the box. Significance levels: * < 0.05, ** < 0.01, *** < 0.001.

A two-way repeated-measures ANOVA with the factors Condition (teaching vs. performing) and Transition Type (FtoP vs. PtoF) showed that there was a significant main effect of Transition Type (*F*(1,19) = 126, *p* < 0.001, ${\eta }_{G}^{2}$ = 0.83) and a significant interaction between Condition and Transition Type (*F*(1,19) = 9.13, *p* = 0.007, ${\eta }_{G}^{2}$ = 0.059, Fig. 9). Post-hoc comparisons based on the estimated marginal means with Tukey adjustment showed that there was a larger KV difference when changing from forte to piano (*p* = 0.028) and from piano to forte (*p* = 0.004) in the teaching condition [FtoP: *M* = − 17.28, *SD* = 8.17, PtoF: *M* = 20.72, *SD* = 8.87] than in the performing condition [FtoP: *M* = − 14.08, *SD* = 7.62, PtoF: *M* = 16.08, *SD* = 7.32].

#### Key-overlap ratios (KORs)

A two-way repeated-measures ANOVA with the factors Condition (teaching vs. performing) and Dynamics (forte vs. piano) showed that there was a significant main effect of Dynamics (*F*(1,19) = 629, *p* < 0.001, ${\eta }_{G}^{2}$ = 0.94), reflecting more key-overlap for forte notes compared to piano notes. Neither the main effect of Condition (*F*(1,19) = 0.084, *p* = 0.77, ${\eta }_{G}^{2}$ = 0.000) nor the interaction between Condition and Dynamics (*F*(1,19) = 0.45, *p* = 0.51, ${\eta }_{G}^{2}$ = 0.001, Fig. 7) was significant.

### Discussion

The results from Experiment 2 replicate our earlier findings and provide further evidence that skilled pianists modulated their performance in specific ways for teaching purposes. As in Experiment 1, we observed slower performance only while teaching the notated articulation. Again, KORs showed that when teaching articulation, participants exaggerated staccato while there was no significant difference in legato between the two conditions. While teaching dynamics, KV showed that participants exaggerated forte and piano. This pattern of exaggeration was more pronounced than in Experiment 1, where post-hoc comparions did not find significant differences between the two conditions for each subcomponent. Furthermore, we again found that when teaching dynamics, at transition points participants produced a larger contrast in dynamics between forte and piano, bidirectionally. Importantly, participants did not modulate performance aspects that were irrelevant for the techniques to be taught (i.e., no modulation of dynamics when teaching articulation and vice versa). Overall, Experiment 2 demonstrated systematic and fine-grained didactic performance modulations for a naturalistic piece of music.

## General discussion

The aim of this study was to investigate how expert pianists modulate their performance to teach techniques necessary to implement musical expressions. Overall, the findings of the two experiments showed that pianists performed one and the same piece differently depending on whether they played with the intention to teach techniques or with the intention to perform the piece for an audience. When playing with the intention to teach, pianists selectively highlighted relevant aspects of artistic expressions. Across two different pieces, participants exaggerated staccato when playing with the intention to teach expressions concerning articulation. The lack of a difference for legato when teaching articulation might stem from a ceiling effect because there might not be enough room for exaggeration without significantly dropping the tempo. When playing with the intention to teach expressions concerning dynamics, participants made larger dynamics changes between forte and piano (in both directions). This constitutes an effective way to highlight the technique as loudness is determined relatively. Taken together, our findings demonstrate that expert pianists systematically modulated their sound depending on the specific technique they were trying to convey.

Although participants tended to play slower in the teaching condition than in the performing condition in general, we found a significant difference only for articulation, not for dynamics. As mentioned in the discussion of Experiment 1, it could be that the metronome beats given prior to each performance discouraged participants from deviating from the prescribed tempo. Another possibility is that expression is best taught when leaving the tempo unchanged when it is irrelevant. This would imply that general didactic modulations like slowing down might be less useful in the context of teaching techniques where timing itself can be used to add an expression to a performance.

One limitation of the study is that although we instructed participants to imagine a situation in which they were teaching musical expression to students, there was no feedback from actual students. It can be expected that teachers would also make didactic modulations in some domains other than acoustic properties, such as gestures if they are physically present in front of students. Moreover, given that teachers modulate their demonstration throughout ongoing interactions with learners^20^, future studies are needed to investigate how experts dynamically adapt their performance to their students’ skill levels and demonstrated abilities. Also, some of the participants in the current study did not have teaching experience at all and even those who had teaching experience had taught for only a few years. It would be important to investigate if our findings would be observed in participants with extensive experience teaching the piano. We summarised descriptive statistics depending on participants’ teaching experience (see Supplementary Material 2).

Another limitation of the study is that it is not certain how the results might generalise to performances of more complex pieces and pieces with fewer notations. In more complex pieces of music, multiple expressive notations could be assigned to one note or phrase. Also, it is rare that almost all the notes are assigned to one expressive notation as in our stimuli. Future studies are needed to determine whether pianists would exaggerate specific parts of a piece or prioritise one aspect over the others given multiple concurrent expressive notations and more possibilities for giving the music different interpretations.

We quantified participants’ sound modulations in terms of tempo, articulation and dynamics so as to examine if and how participants modulate their sound. However, it is possible that participants show didactic modulations in other aspects such as temporal variability and temporal grouping (see Supplementary Materials 3 and 4). For example, participants tended to slow down at transition points regardless of the techniques they were teaching although overall, we found that participants played slower only when teaching the piece with the notated articulation. Future research is needed to investigate how musicians make particular modulations in relation to musical structures.

Musicians and music teachers tend to consider expressive skills as performers’ most important skills^21^. In the present study, experts focused on the teaching of specific expressions that were notated. However, expression also has other facets^22^ that are not only piece-related (e.g., notated expressions) but performer- or context-related (e.g., performer’s interpretation, listening contexts such as recordings or concerts). The ultimate goal of expressive performance is considered to lie in developing performers’ own interpretations of music and convey affects and emotions by using expressive tools such as articulation, dynamics, tempo and timing^23^. The current research opens up the possibility of studying the teaching of expressive performance by demonstrating that pianists could signal didactic intentions without relying on verbal instructions, which are heavily used in actual teaching settings^15,24^. Given that students often learn by listening to recordings^25^, it is important to investigate how performance itself could affect students’ learning processes. It would also be interesting to examine how teachers’ playing (i.e., demonstrations) relates to the verbal instructions they give.

The present findings extend earlier research on teaching-related action modulations. First, our study sheds light on the teaching of technical skills that are an integral part of skill acquisition in artistic domains. We showed that compared to an expressive performance baseline, experts made specific modulations to teach particular techniques. Second, the specificity of the observed modulations supports the idea that teaching comprises more than generic modulations like slowing down or overall exaggeration that may draw learners’ attention. Rather, expert demonstrators seem to follow principles of relevance in communication^26^, highlighting only those aspects they are intending to demonstrate. How learners benefit from the perceptual and motor cues that come with specific exaggerations, and whether understanding the teacher’s intentions explicitly adds to the learning success are important questions for future research.

## Supplementary Information

Supplementary Information.

## Data Availability

All data is available at https://osf.io/8nbjh/.
