# Supplementary material for: Expert pianists make specific exaggerations for teaching
Source: Sci Rep. 2022 Dec 9;12:21296. doi: 10.1038/s41598-022-25711-3 (PMC9734145; doi:10.1038/s41598-022-25711-3)
Supplement: Supplementary file 1 — Supplementary Information. [file 41598_2022_25711_MOESM1_ESM.pdf]

Supplementary Material

Expert pianists make specific exaggerations for teaching

Atsuko Tominaga[1], Günther Knoblich[1] and Natalie Sebanz[1]

[1] Department of Cognitive Science, Central European University,

Quellenstraße 51, 1100 Vienna, Austria

Supplementary Material 1: Instructions

Experiment 1

In the teaching condition, participants were shown the following instruction on a computer monitor (Italic sentences were highlighted in yellow colour on a black background): "Now, play what you practised as if you were teaching it to students. Students already know how to produce the sequence of the tones and now are trying to *learn how to perform the piece expressively* by listening to your performance. *Do your best as a teacher to produce the piece according to the notation that you just practised.*"

In the performing condition, participants were shown the following instruction on a computer monitor: "Now, play what you practised as if you were performing it to an audience. *Do your best as a performer to produce the piece according to the notation that you just practised.*"

Experiment 2

In the teaching condition of Experiment 2, we gave participants the exact same instruction as in Experiment 1. In the performing condition, participants were given the following instruction on a computer monitor: "Now, play what you practised as if you were performing it to an audience. *Perform the piece expressively with your interpretation. Do your best as a performer to produce the piece according to the notation that you just practised.*"

Supplementary Material 2:

Descriptive statistics depending on participants' teaching experience

We summarised descriptive statistics for those with and without teaching experience in piano. Due to the lack of statistical power, we did not perform any statistical tests.

Experiment 1

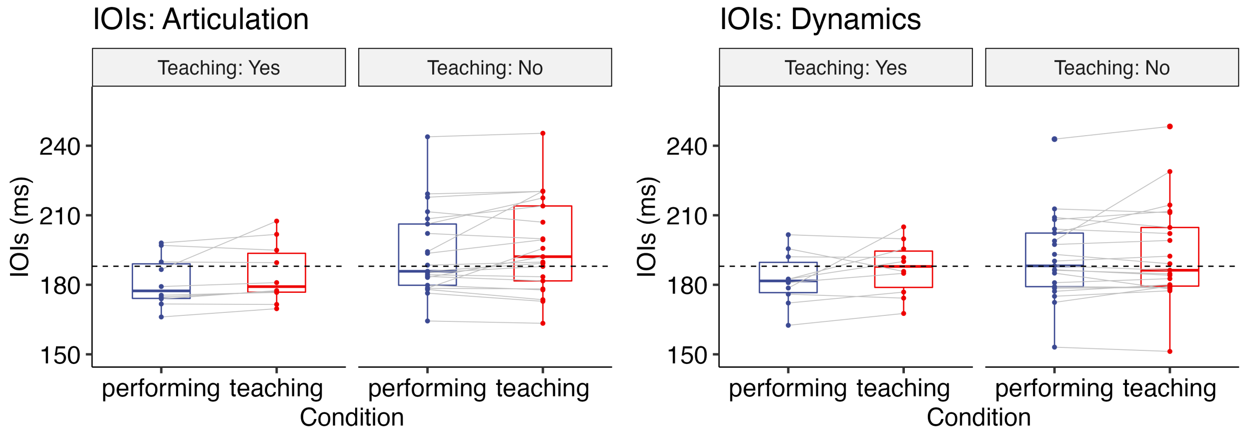

Figure S1. Experiment 1: Comparison between those with and without piano teaching experience in terms of IOIs (ms) when playing the piece with either articulation (left) or dynamics (right). A dashed line represents the tempo given by a metronome. Each box indicates the IQR with the median, and whiskers extend to a maximum of 1.5 × IQR beyond the box.

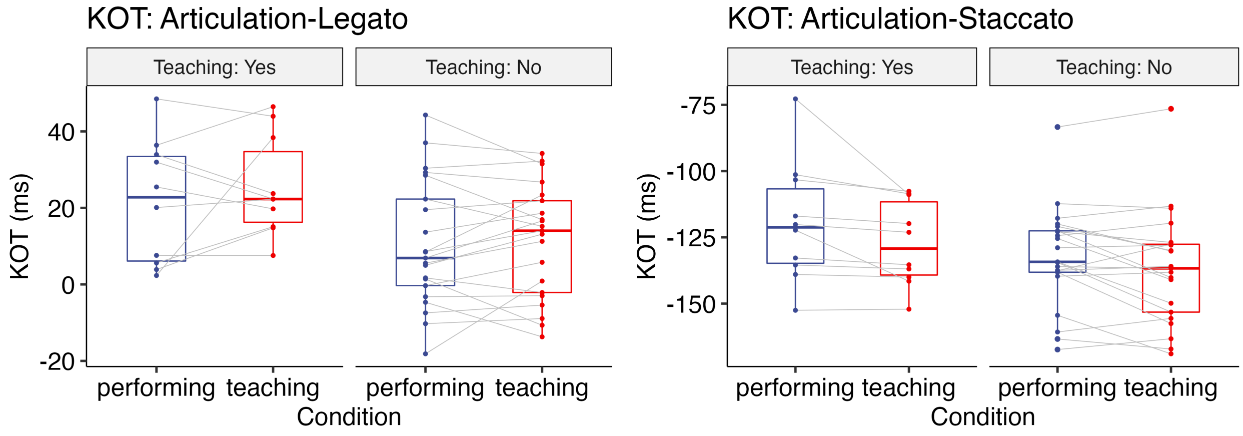

Figure S2. Experiment 1: Comparison between those with and without piano teaching experience in terms of KOT (ms) when playing the piece with articulation (left: legato, right: staccato). Each box indicates the IQR with the median, and whiskers extend to a maximum of 1.5 × IQR beyond the box.

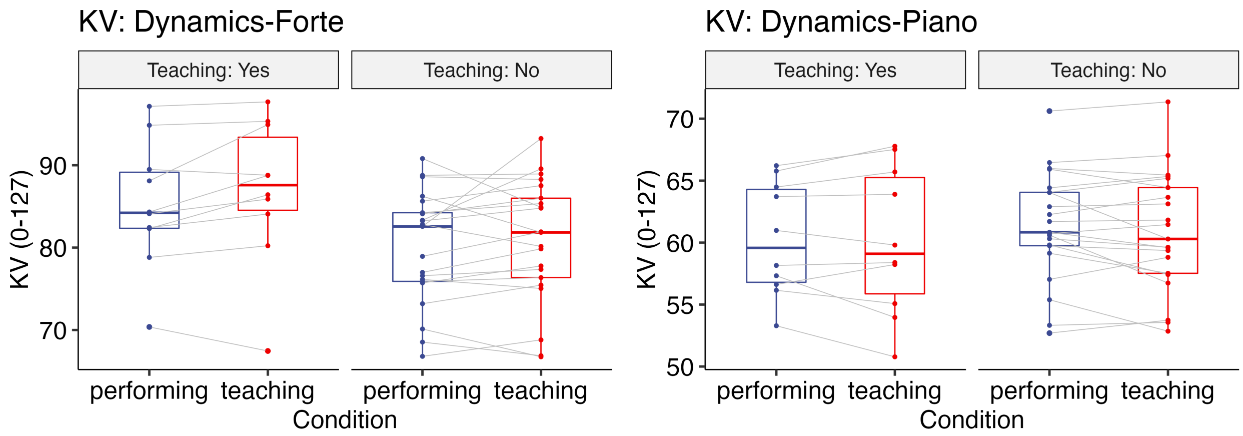

Figure S3. Experiment 1: Comparison between those with and without piano teaching experience in terms of KV (0-127) when playing the piece with dynamics (left: forte, right: piano). Each box indicates the IQR with the median, and whiskers extend to a maximum of 1.5 × IQR beyond the box.

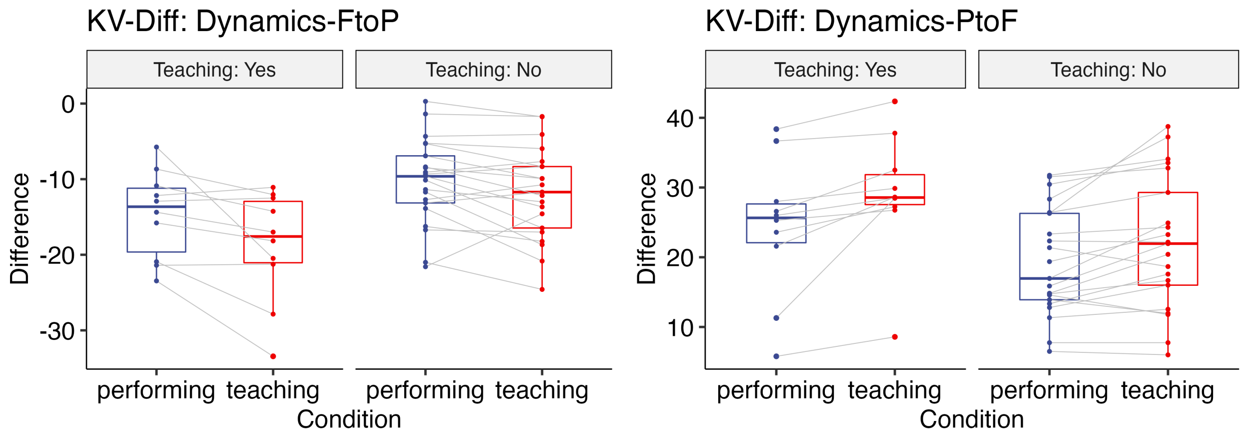

Figure S4. Experiment 1: Comparison between those with and without piano teaching experience in terms of KV Difference at transition points when playing the piece with dynamics (left: forte to piano, right: piano to forte). Each box indicates the IQR with the median, and whiskers extend to a maximum of 1.5 × IQR beyond the box.

Table S1:

Experiment 1: Comparison between those with and without piano teaching experience in terms of IOIs (ms).

Condition	Technique	TeachingExp	N	Mean	SD	SEM
Performing	Articulation	Yes	10	181.27	10.99	3.47
Teaching	Articulation	Yes	10	184.72	13.02	4.12
Performing	Articulation	No	21	193.49	18.75	4.09
Teaching	Articulation	No	21	196.67	20.52	4.48
Performing	Dynamics	Yes	10	182.43	11.52	3.64
Teaching	Dynamics	Yes	10	187.15	11.73	3.71
Performing	Dynamics	No	21	191.48	18.56	4.05
Teaching	Dynamics	No	21	193.50	21.22	4.63

Note. N, SD and SEM represent sample size, standard deviation and standard error of the mean, respectively. Technique indicates performance data when participants playing the piece with either articulation (Fig.1 B) or dynamics (Fig.1 C). TeachingExp indicates if participants had experience in teaching the piano.

Table S2:

Experiment 1: Comparison between those with and without piano teaching experience in terms of KOT (ms).

Condition	Technique	Subcomponent	TeachingExp	N	Mean	SD	SEM
Performing	Articulation	Legato	Yes	10	21.57	16.18	5.12
Teaching	Articulation	Legato	Yes	10	25.43	13.11	4.15
Performing	Articulation	Staccato	Yes	10	-119.66	22.84	7.22
Teaching	Articulation	Staccato	Yes	10	-127.39	15.95	5.04
Performing	Articulation	Legato	No	21	10.40	16.57	3.62
Teaching	Articulation	Legato	No	21	11.36	14.71	3.21
Performing	Articulation	Staccato	No	21	-132.48	19.03	4.15
Teaching	Articulation	Staccato	No	21	-136.84	21.52	4.70

Note. N, SD and SEM represent sample size, standard deviation and standard error of the mean, respectively. Technique indicates performance data when participants play the piece with either articulation (Fig.1 B) or dynamics (Fig.1 C). TeachingExp indicates if participants had experience in teaching the piano.

Table S3:

Experiment 1: Comparison between those with and without piano teaching experience in terms of KV (0-127).

Condition	Technique	Subcomponent	TeachingExp	N	Mean	SD	SEM
Performing	Dynamics	Forte	Yes	10	85.20	7.76	2.45
Teaching	Dynamics	Forte	Yes	10	86.96	8.76	2.77
Performing	Dynamics	Piano	Yes	10	60.27	4.57	1.44
Teaching	Dynamics	Piano	Yes	10	60.11	5.93	1.87
Performing	Dynamics	Forte	No	21	79.97	6.82	1.49
Teaching	Dynamics	Forte	No	21	80.80	7.49	1.63
Performing	Dynamics	Piano	No	21	61.33	4.40	0.96
Teaching	Dynamics	Piano	No	21	60.88	4.76	1.04

Note. N, SD and SEM represent sample size, standard deviation and standard error of the mean, respectively. Technique indicates performance data when participants play the piece with either articulation (Fig.1 B) or dynamics (Fig.1 C). TeachingExp indicates if participants had experience in teaching the piano.

Table S4:

Experiment 1: Comparison between those with and without piano teaching experience in terms of KV Difference at transition points.

Condition	Technique	Subcomponent	TeachingExp	N	Mean	SD	SEM
Performing	Dynamics	FtoP	Yes	10	-14.63	5.79	1.83
Teaching	Dynamics	FtoP	Yes	10	-18.81	7.26	2.30
Performing	Dynamics	PtoF	Yes	10	24.32	9.96	3.15
Teaching	Dynamics	PtoF	Yes	10	29.07	8.78	2.78
Performing	Dynamics	FtoP	No	21	-10.30	5.69	1.24
Teaching	Dynamics	FtoP	No	21	-11.87	6.09	1.33
Performing	Dynamics	PtoF	No	21	19.23	7.71	1.68
Teaching	Dynamics	PtoF	No	21	21.98	9.56	2.09

Note. N, SD and SEM represent sample size, standard deviation and standard error of the mean, respectively. Technique indicates performance data when participants play the piece with either articulation (Fig.1 B) or dynamics (Fig.1 C). TeachingExp indicates if participants had experience in teaching the piano.

Experiment 2

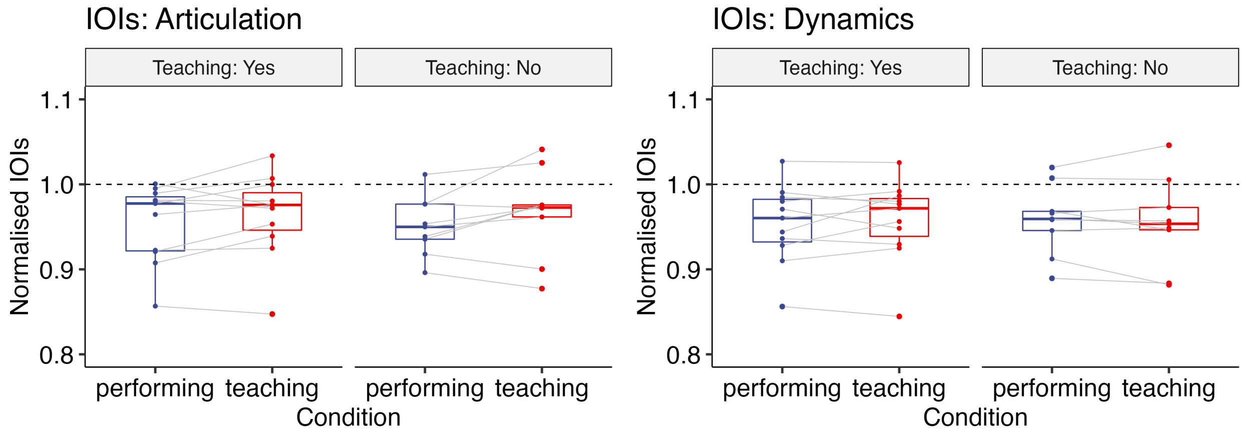

Figure S5. Experiment 2: Comparison between those with and without piano teaching experience in terms of normalised IOIs when playing the piece with either articulation (left) or dynamics (right). A dashed line represents the tempo given by a metronome. Each box indicates the IQR with the median, and whiskers extend to a maximum of 1.5 × IQR beyond the box.

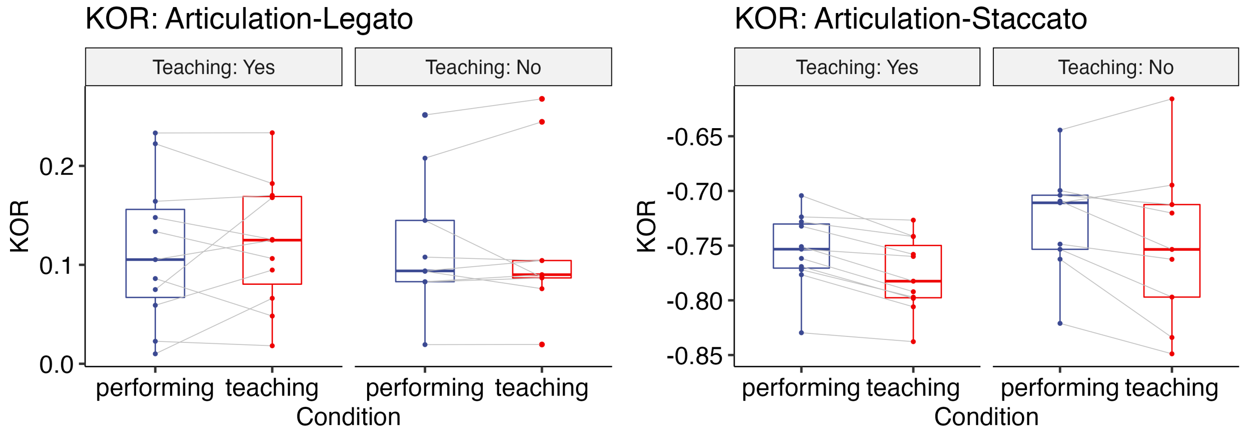

Figure S6. Experiment 2: Comparison between those with and without piano teaching experience in terms of KOR when playing the piece with articulation (left: legato, right: staccato). Each box indicates the IQR with the median, and whiskers extend to a maximum of 1.5 × IQR beyond the box.

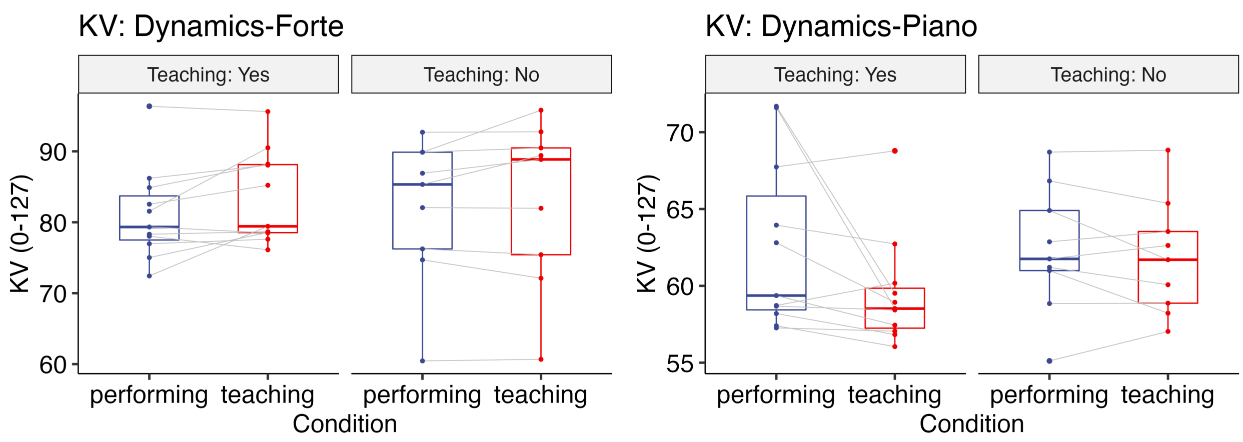

Figure S7. Experiment 2: Comparison between those with and without piano teaching experience in terms of KV (0-127) when playing the piece with dynamics (left: forte, right: piano). Each box indicates the IQR with the median, and whiskers extend to a maximum of 1.5 × IQR beyond the box.

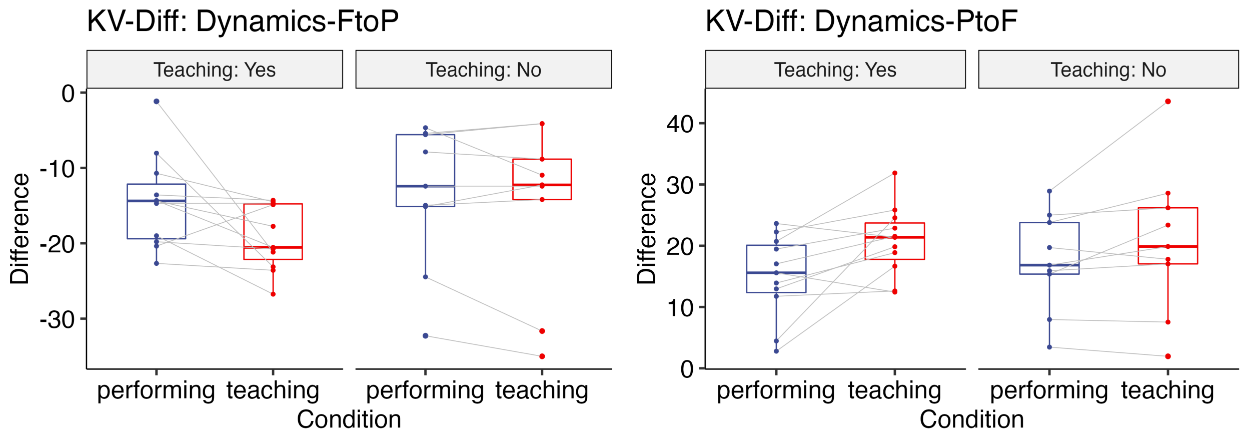

Figure S8. Experiment 2: Comparison between those with and without piano teaching experience in terms of KV Difference at transition points when playing the piece with dynamics (left: forte to piano, right: piano to forte). Each box indicates the IQR with the median, and whiskers extend to a maximum of 1.5 × IQR beyond the box.

Table S5:

Experiment 2: Comparison between those with and without piano teaching experience in terms of normalised IOIs.

Condition	Technique	TeachingExp	N	Mean	SD	SEM
Performing	Articulation	Yes	11	0.954	0.046	0.014
Teaching	Articulation	Yes	11	0.964	0.049	0.015
Performing	Articulation	No	9	0.951	0.035	0.012
Teaching	Articulation	No	9	0.967	0.052	0.017
Performing	Dynamics	Yes	11	0.953	0.046	0.014
Teaching	Dynamics	Yes	11	0.958	0.047	0.014
Performing	Dynamics	No	9	0.958	0.041	0.014
Teaching	Dynamics	No	9	0.955	0.052	0.017

Note. N, SD and SEM represent sample size, standard deviation and standard error of the mean, respectively. Technique indicates performance data when participants play the piece with either articulation (Fig.5 B) or dynamics (Fig.5 C). TeachingExp indicates if participants had experience in teaching the piano.

Table S6:

Experiment 2: Comparison between those with and without piano teaching experience in terms of KORs.

Condition	Technique	Subcomponent	TeachingExp	N	Mean	SD	SEM
Performing	Articulation	Legato	Yes	11	0.115	0.074	0.022
Teaching	Articulation	Legato	Yes	11	0.122	0.064	0.019
Performing	Articulation	Staccato	Yes	11	-0.755	0.034	0.010
Teaching	Articulation	Staccato	Yes	11	-0.776	0.034	0.010
Performing	Articulation	Legato	No	9	0.120	0.071	0.024
Teaching	Articulation	Legato	No	9	0.120	0.081	0.027
Performing	Articulation	Staccato	No	9	-0.728	0.050	0.017
Teaching	Articulation	Staccato	No	9	-0.749	0.073	0.024

Note. N, SD and SEM represent sample size, standard deviation and standard error of the mean, respectively. Technique indicates performance data when participants play the piece with either articulation (Fig.5 B) or dynamics (Fig.5 C). TeachingExp indicates if participants had experience in teaching the piano.

Table S7:

Experiment 2: Comparison between those with and without piano teaching experience in terms of KV (0-127).

Condition	Technique	Subcomponent	TeachingExp	N	Mean	SD	SEM
Performing	Dynamics	Forte	Yes	11	81.06	6.52	1.97
Teaching	Dynamics	Forte	Yes	11	83.32	6.48	1.95
Performing	Dynamics	Piano	Yes	11	62.49	5.55	1.67
Teaching	Dynamics	Piano	Yes	11	59.50	3.59	1.08
Performing	Dynamics	Forte	No	9	82.02	10.15	3.38
Teaching	Dynamics	Forte	No	9	83.06	11.54	3.85
Performing	Dynamics	Piano	No	9	62.36	4.11	1.37
Teaching	Dynamics	Piano	No	9	61.81	3.76	1.25

Note. N, SD and SEM represent sample size, standard deviation and standard error of the mean, respectively. Technique indicates performance data when participants play the piece with either articulation (Fig.5 B) or dynamics (Fig.5 C). TeachingExp indicates if participants had experience in teaching the piano.

Table S8:

Experiment 2: Comparison between those with and without piano teaching experience in terms of KV Difference at transition points.

Condition	Technique	Subcomponent	TeachingExp	N	Mean	SD	SEM
Performing	Dynamics	FtoP	Yes	11	-14.43	6.20	1.87
Teaching	Dynamics	FtoP	Yes	11	-19.27	4.33	1.30
Performing	Dynamics	PtoF	Yes	11	14.96	6.78	2.04
Teaching	Dynamics	PtoF	Yes	11	20.77	5.71	1.72
Performing	Dynamics	FtoP	No	9	-13.64	9.46	3.15
Teaching	Dynamics	FtoP	No	9	-14.84	11.08	3.69
Performing	Dynamics	PtoF	No	9	17.44	8.11	2.70
Teaching	Dynamics	PtoF	No	9	20.66	12.09	4.03

Note. N, SD and SEM represent sample size, standard deviation and standard error of the mean, respectively. Technique indicates performance data when participants play the piece with either articulation (Fig.5 B) or dynamics (Fig.5 C). TeachingExp indicates if participants had experience in teaching the piano.

Supplementary Material 3: Temporal Variability

The tempo variability of performance was assessed with the coefficient of variation (CV) of the IOIs to investigate the tempo stability of performance between the teaching and performing condition. CVs were calculated by dividing the standard deviation by the mean of IOIs.

Results

Experiment 1

Articulation. To compare the mean CVs between the teaching and performing condition, we conducted a Wilcoxon Signed-rank test, instead of a paired *t*-test, because a Shapiro-Wilk test showed that the distribution of the mean difference was significantly different from the normal distribution ($p = 0.007$). The Wilcoxon Signed-rank test showed that the tempo variability of performance did not differ significantly between the teaching [$Mdn = 0.064$, $IQR = 0.024$] and performing condition [$Mdn = 0.066$, $IQR = 0.016$] while playing the piece with the notated articulation ($p = 0.06$, $r = 0.34$, two-tailed, *Fig S9*).

Dynamics. To compare the mean CVs between the teaching and performing condition, we conducted a Wilcoxon Signed-rank test, instead of a paired *t*-test, because a Shapiro-Wilk test showed that the distribution of the mean difference was significantly different from the normal distribution ($p = 0.024$). The Wilcoxon Signed-rank test showed that the tempo variability of performance did not differ significantly between the teaching [$Mdn = 0.070$, $IQR = 0.013$] and performing condition [$Mdn = 0.074$, $IQR = 0.026$] while playing the piece with the notated dynamics ($p = 0.22$, $r = 0.22$, two-tailed, *Fig S9*).

Experiment 2

Articulation. To compare the mean CVs between the teaching and performing condition, we conducted a Wilcoxon Signed-rank test, instead of a paired *t*-test, because a Shapiro-Wilk test showed that the distribution of the mean difference was significantly different from the normal distribution (p = 0.017). The Wilcoxon Signed-rank test showed that the tempo variability of performance did not differ significantly between the teaching [Mdn = 0.044, IQR = 0.012] and performing condition [Mdn = 0.047, IQR = 0.014] while playing the piece with the notated articulation (p = 0.07, r = 0.41, two-tailed, *Fig S9*).

Dynamics. A paired-sample *t*-test showed that the tempo variability of performance did not differ significantly between the teaching [M = 0.048, SD = 0.011] and the performing condition [M = 0.048, SD = 0.011] while playing the piece with the notated dynamics ($t(19)$ = 1.83, p = 0.08, Cohen's d = 0.41, two-tailed, *Fig S9*).

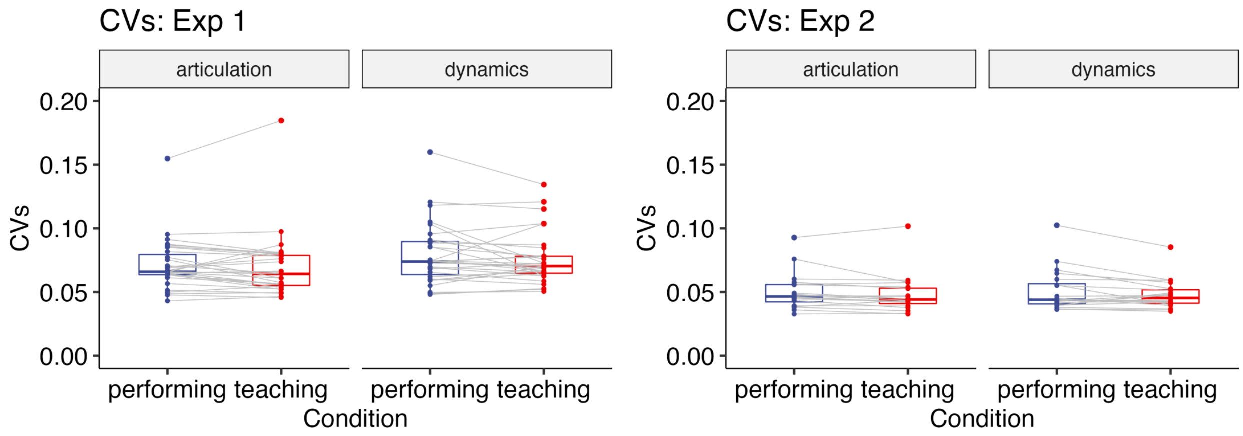

Figure S9. Experiment 1, 2: CVs when playing the piece with either articulation or dynamics (left: Experiment 1, right: Experiment 2). Each box indicates the IQR with the median, and whiskers extend to a maximum of 1.5 × IQR beyond the box. Significance levels: * < .05, ** < .01, *** < .001

Supplementary Material 4: Temporal grouping

In order to investigate if participants used temporal grouping to make boundaries between subcomponents (i.e., between legato and staccato, between forte and piano) clearer for teaching purposes, we looked at IOIs only at transition points.

Results

Experiment 1

Articulation. To compare the mean IOIs at transition points between the teaching and performing condition, we conducted a Wilcoxon Signed-rank test, instead of a paired *t*-test, because a Shapiro-Wilk test showed that the distribution of the mean difference was significantly different from the normal distribution ($p < 0.001$). The Wilcoxon Signed-rank test revealed that IOIs at transition points were larger in the teaching condition [$Mdn = 195.75$ (ms), $IQR = 25.75$] than in the performing condition [$Mdn = 188.77$ (ms), $IQR = 23.53$] while playing the piece with the notated articulation ($p = 0.02$, $r = 0.42$, two-tailed, *Fig S10*).

Dynamics. A paired-sample *t*-test showed that IOIs at transition points were larger in the teaching condition [$M = 200.90$ (ms), $SD = 20.96$] than in the performing condition [$M = 197.63$ (ms), $SD = 19.30$] while playing the piece with the notated dynamics ($t(30) = 2.10$, $p = 0.04$, Cohen's $d = 0.38$, two-tailed, *Fig S10*).

Experiment 2

Articulation. A paired-sample *t*-test showed that IOIs at transition points were larger in the teaching condition [*M* = 1.00, *SD* = 0.067] than in the performing condition [*M* = 0.98, *SD* = 0.049] while playing the piece with the notated articulation ($t(19) = 2.27$, $p = 0.03$, Cohen's $d = 0.51$, two-tailed, *Fig S10*).

Dynamics. To compare the mean IOIs at transition points between the teaching and performing condition, we conducted a Wilcoxon Signed-rank test, instead of a paired *t*-test, because a Shapiro-Wilk test showed that the distribution of the mean difference was significantly different from the normal distribution ($p = 0.004$). The Wilcoxon Signed-rank test revealed that IOIs at transition points did not differ significantly between the teaching [*Mdn* = 1.00, *IQR* = 0.06] and the performing condition [*Mdn* = 0.99, *IQR* = 0.05] while playing the piece with the notated dynamics ($p = 0.60$, $r = 0.13$, two-tailed, *Fig S10*).

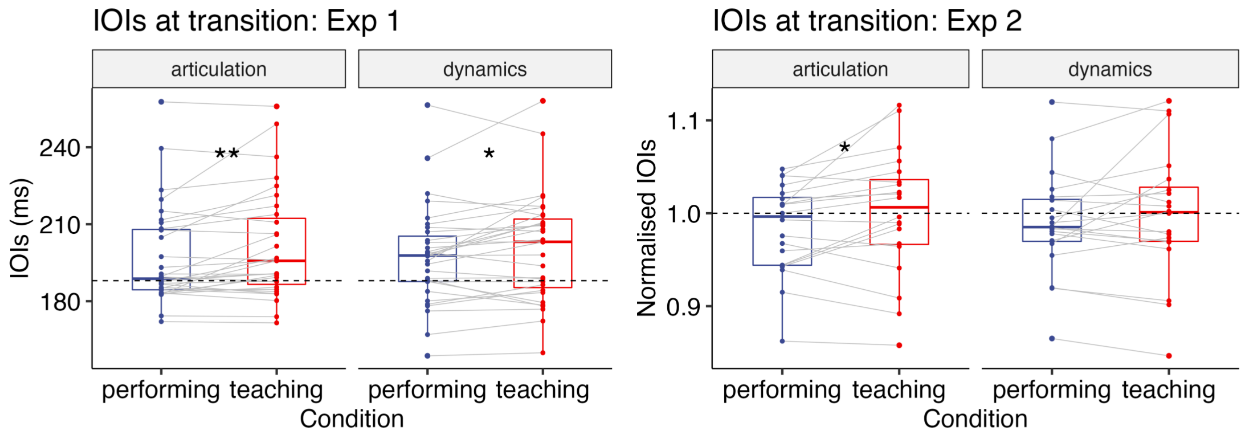

Figure S10. Experiment 1, 2: IOIs at transition points when playing the piece with either articulation or dynamics (left: Experiment 1, right: Experiment 2). A dashed line represents the tempo given by a metronome. Each box indicates the IQR with the median, and whiskers extend to a maximum of 1.5 × IQR beyond the box. Significance levels: * < .05, ** < .01, *** < .001

Supplementary Material 5: Additional analysis for dynamics difference

In order to examine if the dynamics difference between forte and piano at transition points is larger when the difference between forte and piano in overall performance is considered, we normalised the KV difference at transition points by dividing it by the average range between forte and piano per performance (per trial).

Results

Experiment 1

A two-way repeated-measures ANOVA with the factors Condition (teaching vs. performing) and Transition Type (FtoP vs. PtoF) showed that there was a significant main effect of Transition Type ($F(1,30) = 741$, $p < 0.001$, $\eta^2_G = 0.88$) and a significant interaction between Condition and Transition Type ($F(1,30) = 7.87$, $p = 0.009$, $\eta^2_G = 0.009$, *Fig S11*). Post-hoc comparisons based on the estimated marginal means with Tukey adjustment showed that there was no significant difference when changing from forte to piano ($p = 0.085$) and from piano to forte ($p = 0.075$) between the teaching condition [FtoP: $M = -0.62$, $SD = 0.24$, PtoF: $M = 1.10$, $SD = 0.34$] and performing condition [FtoP: $M = -0.56$, $SD = 0.25$, PtoF: $M = 1.03$, $SD = 0.40$].

Experiment 2

A two-way repeated-measures ANOVA with the factors Condition (teaching vs. performing) and Transition Type (FtoP vs. PtoF) showed that there was a significant main effect of Transition Type ($F(1,19) = 42$, $p < 0.001$, $\eta^2_G = 0.63$). However, there was no significant interaction between Condition and Transition Type ($F(1,19) = 1.10$, $p = 0.31$, $\eta^2_G = 0.008$, *Fig S11*).

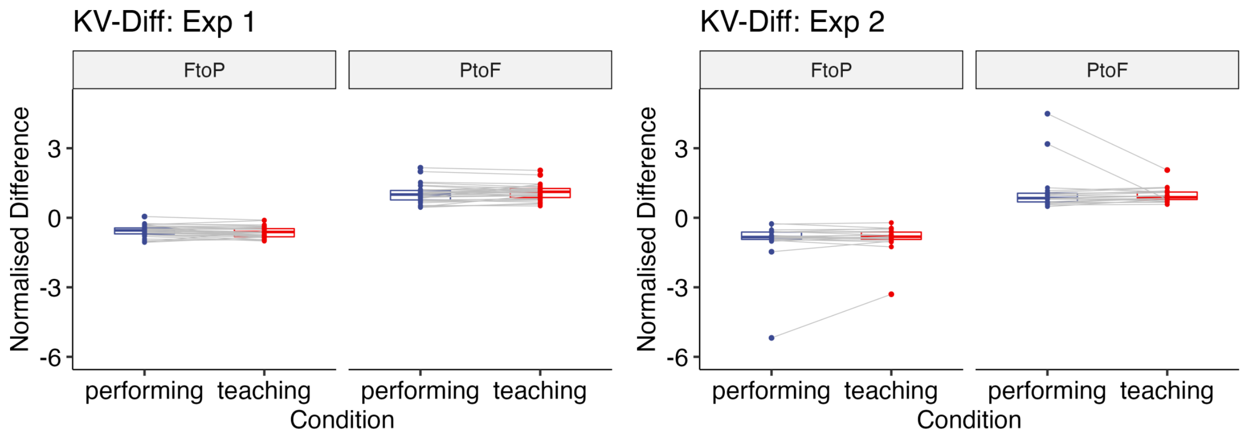

Figure S11. Experiment 1, 2: Normalised KV Difference at transition points when playing the piece with dynamics (left: Experiment 1, right: Experiment 2). Each box indicates the IQR with the median, and whiskers extend to a maximum of 1.5 × IQR beyond the box. Significance levels: * < .05, ** < .01, *** < .001
